# 1,4-Dihydro­benzo[*g*]quinoxaline-2,3-dione

**DOI:** 10.1107/S1600536812047526

**Published:** 2012-11-28

**Authors:** François Eya’ane Meva, Dieter Schaarschmidt, Mohammad A. Abdulmalic, Tobias Rüffer

**Affiliations:** a Department of Pharmaceutical Sciences, Faculty of Medicine and Pharmaceutical Sciences, University of Douala, BP 2701, Cameroon; b Technische Universität Chemnitz, Fakultät für Naturwissenschaften, Institut für Chemie, Lehrstuhl für Anorganische Chemie, Strasse der Nationen 62, 09111 Chemnitz, Germany

## Abstract

The title compound, C_12_H_8_N_2_O_2_, was prepared by the reaction of the diethyl ester of naphthalene­bis­(oxamate) with *tert*-BuNH_2_. The mol­ecule is nearly planar, with an r.m.s. deviation of 0.017 Å from the plane through all 16 non-H atoms. In the crystal, a three-dimensional network is formed, composed of layers of mol­ecules along the *b*- and *c*-axis directions, due to the formation of inter­molecular N—H⋯O hydrogen bonds, as well as of chains along the *a-*axis direction due to parallel displaced sandwich-type π–π inter­actions with average distances between the inter­acting mol­ecules in the range 3.35–3.40 Å.

## Related literature

For the synthesis and structure of 1,4-dihydro­benzo[*g*]quin­oxaline-2,3-dione·3H_2_O, see: Oxtoby *et al.* (2005 ▶). For the use of bis­(oxamates) and bis­(oxamidates) for complex formation, see: Pardo *et al.* (2008 ▶) and Abdulmalic *et al.* (2012 ▶); Rüffer *et al.* (2012 ▶), respectively. For the general synthesis of bis­(oxamidates), see: Ruiz *et al.* (1997 ▶) and for the synthesis of diethyl *N,N*’-naphtalene-bis­(oxamate), see: Rüffer *et al.* (2007 ▶). For thin film formation by bis­(oxamato) complexes, see: Eya’ane Meva (2009 ▶); Bräuer *et al.* (2006 ▶). For self-organization in supra­molecular chemistry due to inter­molecular π inter­actions and/or hydrogen bonds, see: Burrow *et al.* (1996 ▶); Chowdhry *et al.* (1996 ▶); Dai *et al.* (1997 ▶); Munoz *et al.* (1998 ▶). For dione tautomerism in the solid state, see: Svenson (1976 ▶).
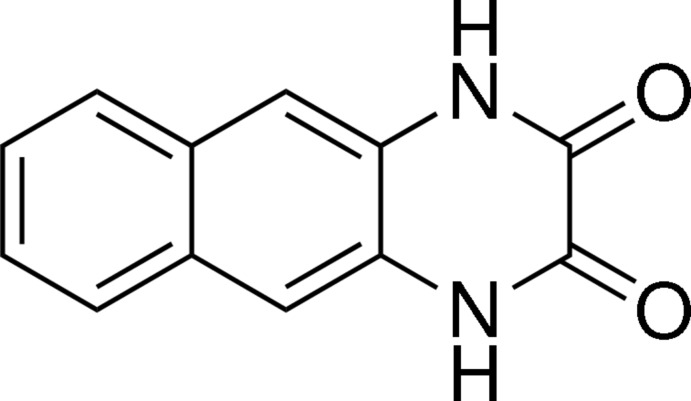


## Experimental

### 

#### Crystal data

C_12_H_8_N_2_O_2_
*M* *_r_* = 212.20
Monoclinic, 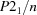

*a* = 7.1334 (15) Å
*b* = 8.4229 (18) Å
*c* = 15.292 (2) Å
β = 99.792 (14)°
*V* = 905.4 (3) Å^3^
*Z* = 4
Mo *K*α radiation
μ = 0.11 mm^−1^
*T* = 293 K
0.3 × 0.2 × 0.1 mm

#### Data collection

Oxford Gemini S diffractometer
Absorption correction: multi-scan (*CrysAlis RED*; Oxford Diffraction, 2006 ▶) *T*_min_ = 0.649, *T*_max_ = 1.000
5294 measured reflections
1773 independent reflections
875 reflections with *I* > 2σ(*I*)
*R*_int_ = 0.039

#### Refinement

*R*[*F* ^2^ > 2σ(*F* ^2^)] = 0.094
*wR*(*F* ^2^) = 0.271
*S* = 0.92
1773 reflections
153 parameters
H atoms treated by a mixture of independent and constrained refinement
Δρ_max_ = 0.77 e Å^−3^
Δρ_min_ = −0.57 e Å^−3^

### 

Data collection: *CrysAlis CCD* (Oxford Diffraction, 2006 ▶); cell refinement: *CrysAlis CCD*; data reduction: *CrysAlis RED* (Oxford Diffraction, 2006 ▶); program(s) used to solve structure: *SHELXS97* (Sheldrick, 2008 ▶); program(s) used to refine structure: *SHELXL97* (Sheldrick, 2008 ▶); molecular graphics: *ORTEP-3 for Windows* (Farrugia, 2012 ▶); software used to prepare material for publication: *WinGX* (Farrugia, 2012 ▶) and *publCIF* (Westrip, 2010 ▶).

## Supplementary Material

Crystal structure: contains datablock(s) I, global. DOI: 10.1107/S1600536812047526/sj5284sup1.cif

Structure factors: contains datablock(s) I. DOI: 10.1107/S1600536812047526/sj5284Isup3.hkl

Supplementary material file. DOI: 10.1107/S1600536812047526/sj5284Isup3.cml

Additional supplementary materials:  crystallographic information; 3D view; checkCIF report

## Figures and Tables

**Table 1:** Hydrogen-bond geometry (Å, °)

*D*—H⋯*A*	*D*—H	H⋯*A*	*D*⋯*A*	*D*—H⋯*A*
N1—H1*N*⋯O1^i^	0.91 (4)	2.27 (4)	2.866 (3)	122 (3)
N2—H2*N*⋯O2^ii^	0.94 (4)	1.90 (4)	2.843 (4)	176 (4)

Symmetry codes: (i) 
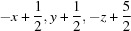
; (ii) 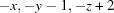.

**Fig. 1.**
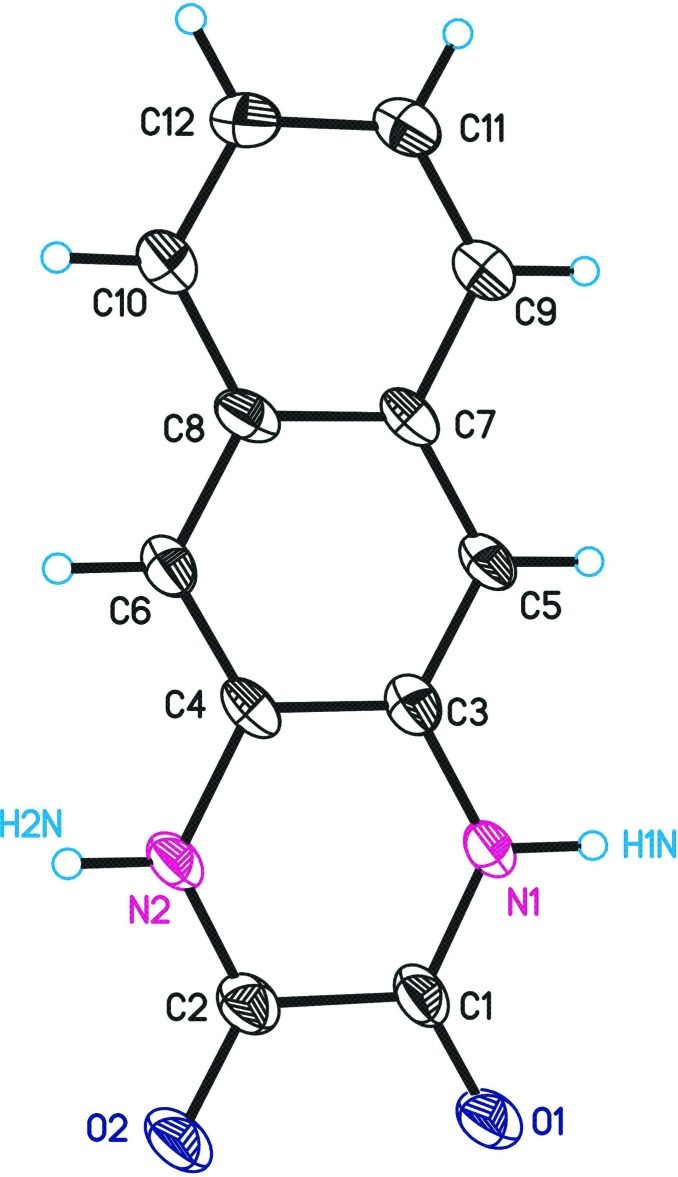
ORTEP diagram (50% ellipsoid probability) of the molecular structure of 1.

**Fig. 2.**
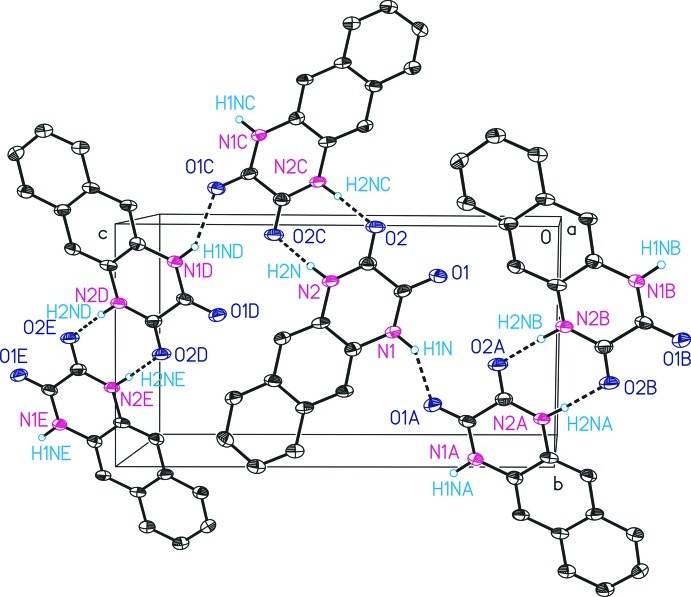
Graphical representation of a part of one two-dimensional layer formed by 1 in the solid state due to formation of intermolecular hydrogen bonds along the crystallographic b- and c-axes. All C-bonded hydrogen atoms are omitted for clarity. Symmetry codes: (A) -3/2 – x, 1/2 + y, 1/2 – z. (B) -1/2 + x, 1/2 – y, -1/2 + z. (C) -1 – x, – y, 1 – z. (D) -1/2 + x, 1/2 – y, 1/2 + z. (E) -1/2 – x, 1/2 + y, 3/2 – z.

**Fig. 3.**
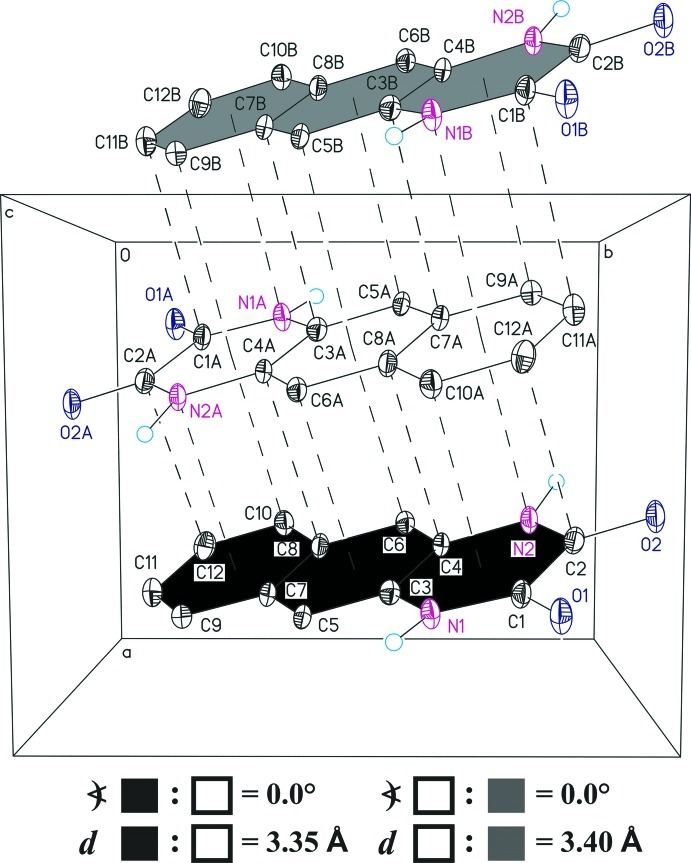
Graphical representation of the parallel-displaced sandwich type π interactions of 1 in the solid state, giving rise to the formation of one-dimensional chains along the crystallographic a-axes. All C-bonded hydrogen atoms are omitted for clarity. The sign &angsph; refers to the interplanar angle and d gives the averaged distances between interacting molecules. Dotted lines indicate interacting C···C atoms as well as interactions between carbon and nitrogen atoms, respectively, with the centroids of the respective adjacent aromatic rings. Symmetry codes: (A) 1 – x, 1 – y, 1 – z. (B) -1 + x, y, z.

**Fig. 4.**
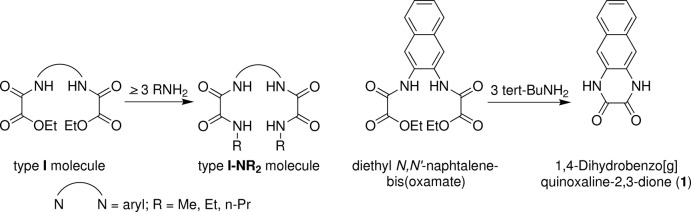
Synthesis of 1.

## supplementary crystallographic information

### Comment

Weak interactions such as intermolecular π interactions or the formation of hydrogen bonds are essential for molecular recognition and self organization in biological systems and supramolecular chemistry (Burrow *et al.*, 1996; Chowdhry *et al.*, 1996; Dai *et al.*, 1997). Oxtoby *et al.* (2005) synthesized derivatives of dihydroquinoxalinedione containing a hydrophilic oxalamide-based "terminus". This oxalamide serves to increase the water solubility of the organic molecules and allows H_2_O molecules to be hydrogen bonded to the organic molecules as well as to each other. In this perspective, 1,4 dihydrobenzo[*g*]quinoxaline-2,3-dione hydrate (1×3H_2_O) has been crystallized by slowly cooling a boiling solution of the powder in DMF/water to room temperature (Oxtoby *et al.*, 2005). Infinite arrays containing two different alternating head-to-tail π interactions parallel to the crystallographic *a* axis were observed in the solid state of 1×3H_2_O, with the π stacks being orthogonal to chains of H_2_O molecules and held together by *R*^2^_2_(8) hydrogen-bonding interactions.

Bis(oxamate) molecules (type I molecules, *cf.* Figure 4) have been widely used in order to produce a series of complexes with various magnetic interactions (Pardo *et al.*, 2008). In previous work, our interest was devoted to a study of the impact of π interactions in the formation of thin films using trimetallic bis(oxamato) type complexes (Eya'ane Meva, 2009; Bräuer *et al.*, 2006) as well as the investigation of electronic effects on magnetic *J* couplings (Rüffer *et al.*, 2007). With the aim of using type I—N*R*_2_ molecules as ligands for the formation of transition metal complexes (Abdulmalic *et al.*, 2012; Rüffer *et al.*, 2012), we became interested in their synthesis. Generally, type I molecules are reacted with an excess of a primary amine (Ruiz *et al.*, 1997) resulting in the formation of type I—N*R*_2_ molecules. On the other hand, quinoxaline derivatives are generally synthesized by refluxing diamines and oxalic acid in HCl (Oxtoby *et al.*, 2005). We report here, that the reaction of the diethyl ester of naphtalene-bis(oxamate) (Rüffer *et al.*, 2007), Fig. 4, with an excess of *tert*-BuNH_2_ in MeOH does not give the corresponding type I—N*R*_2_ molecule, but instead forms 1,4-dihydrobenzo[*g*]quinoxaline-2,3-dione (1). A similar reaction has been already described by Munoz *et al.* (1998), who treated *o*-phenylenebis(oxamate) with [Me_4_N]OH and Fe(ClO_4_)_3_ to obtain an analogous derivatized product in the form of its Fe(III) complex.

In the solid state 1 is nearly planar (r. m. s. d. of a calculated mean plane of C1–C12, O1, O2, N1, N2 = 0.017 Å). The bond lengths of the C═O functions of 1 (1.230 (4) and 1.240 (4) Å) reveal the molecule to be the dione tautomer in the solid state (Svenson, 1976). All other bond distances and angles are comparable those described for 1×3H_2_O (Oxtoby *et al.*, 2005).

In the crystal structure of 1 the formation of a three-dimensional network is observed. Intermolecular hydrogen bonds between individual molecules of 1, *cf.* Table 1, form two-dimensional layers. A representative view of one selected two-dimensional layer is illustrated in Figure 2, showing the two-dimensional layers extending along the crystallographic *b*- and *c*-axes. Within the two-dimensional layers the formation of dimers of 1 with *R*^2^_2_(8) type hydrogen bond interactions is also observed, as reported for 1×3H_2_O (Oxtoby *et al.*, 2005), *cf.* N2—H2*N*···O2^ii^ in Table 1 and Figure 2. However, due to the N1—H1*N*···O1^i^, hydrogen bond *cf.* Table 1, the dimers are connected further to form the two-dimensional layers.

Additionally, individual molecules of 1 interact with each other by means of π interactions. They are approximately arranged in a parallel-displaced sandwich type configuration and thus form one-dimensional layers along the crystallographic *a*-axis, *cf.* Figure 3. Within such a one-dimensional chain, a head-to-tail arrangement is observed, as reported previously for 1×3H_2_O (Oxtoby *et al.*, 2005). Moreover, by analogy with 1×3H_2_O, the π stacks of 1 are arranged orthogonal to the two-dimensional layers formed by intermolecular hydrogen bonds. The combinations of both supramolecular arrangements finally give rise to a three-dimensional network structure.

### Experimental

Diethyl *N,N'*-naphthalene-bis(oxamate) was synthesized according to the literature (Rüffer *et al.*, 2007). To a solution of diethyl naphthalene-bis(oxamate) (1.5 g, 9.49 mmol) in MeOH (50 ml) three equivalents of a solution of *tert*-BuNH_2_ (2.05 g, 28.47 mmol) in MeOH (25 ml) were added. The solution was refluxed for 30 minutes, cooled to room temperature and concentrated to 30 ml. Diethyl ether (100 ml) was added and the resulting brown precipitate was filtered and dried on air. Yellow crystals of 1 were obtained by solvent diffusion of a dilute MeOH solution of 1 against Et_2_O at room temperature. Yield: 0.7 g, 80%.

### Refinement

C-bonded H atoms were placed in calculated positions and constrained to ride on their parent atoms, with a C—H distance of 0.93 Å and *U*_iso_(H) = 1.2*U*_eq_(C). The N-bonded H atoms were located on a difference Fourier map and refined freely. The high R factor, low ratio of observed to unique reflections and relatively high su values indicate that the crystals were not of good quality and were very weakly diffracting.

### Crystal data

**Table:**

C_12_H_8_N_2_O_2_	*F*(000) = 440
*M**_r_* = 212.20	*D*_x_ = 1.557 Mg m^−^^3^
Monoclinic, *P*2_1_/*n*	Mo *K*α radiation, λ = 0.71073 Å
Hall symbol: -P 2yn	Cell parameters from 5135 reflections
*a* = 7.1334 (15) Å	θ = 2.9–25.9°
*b* = 8.4229 (18) Å	µ = 0.11 mm^−^^1^
*c* = 15.292 (2) Å	*T* = 293 K
β = 99.792 (14)°	Block, yellow
*V* = 905.4 (3) Å^3^	0.3 × 0.2 × 0.1 mm
*Z* = 4	

### Data collection

**Table:**

Oxford Gemini S diffractometer	875 reflections with *I* > 2σ(*I*)
Radiation source: fine-focus sealed tube	*R*_int_ = 0.039
Graphite monochromator	θ_max_ = 26.0°, θ_min_ = 3.0°
ω scans	*h* = −8→7
Absorption correction: multi-scan (*CrysAlis RED*; Oxford Diffraction, 2006)	*k* = −10→8
*T*_min_ = 0.649, *T*_max_ = 1.000	*l* = −18→18
5294 measured reflections	2 standard reflections every 50 reflections
1773 independent reflections	intensity decay: none

### Refinement

**Table:**

Refinement on *F*^2^	Primary atom site location: structure-invariant direct methods
Least-squares matrix: full	Secondary atom site location: difference Fourier map
*R*[*F*^2^ > 2σ(*F*^2^)] = 0.094	Hydrogen site location: inferred from neighbouring sites
*wR*(*F*^2^) = 0.271	H atoms treated by a mixture of independent and constrained refinement
*S* = 0.92	*w* = 1/[σ^2^(*F*_o_^2^) + (0.2*P*)^2^] where *P* = (*F*_o_^2^ + 2*F*_c_^2^)/3
1773 reflections	(Δ/σ)_max_ = 0.001
153 parameters	Δρ_max_ = 0.77 e Å^−^^3^
0 restraints	Δρ_min_ = −0.57 e Å^−^^3^

### Special details

**Table:**

Geometry. All e.s.d.'s (except the e.s.d. in the dihedral angle between two l.s. planes) are estimated using the full covariance matrix. The cell e.s.d.'s are taken into account individually in the estimation of e.s.d.'s in distances, angles and torsion angles; correlations between e.s.d.'s in cell parameters are only used when they are defined by crystal symmetry. An approximate (isotropic) treatment of cell e.s.d.'s is used for estimating e.s.d.'s involving l.s. planes.
Refinement. Refinement of *F*^2^ against ALL reflections. The weighted *R*-factor *wR* and goodness of fit *S* are based on *F*^2^, conventional *R*-factors *R* are based on *F*, with *F* set to zero for negative *F*^2^. The threshold expression of *F*^2^ > σ(*F*^2^) is used only for calculating *R*-factors(gt) *etc*. and is not relevant to the choice of reflections for refinement. *R*-factors based on *F*^2^ are statistically about twice as large as those based on *F*, and *R*- factors based on ALL data will be even larger.

### Fractional atomic coordinates and isotropic or equivalent isotropic displacement parameters (Å^2^)

**Table:**

	*x*	*y*	*z*	*U*_iso_*/*U*_eq_	
C1	0.2609 (4)	−0.2869 (4)	1.1809 (2)	0.0276 (8)	
C2	0.1631 (4)	−0.3842 (4)	1.1026 (2)	0.0276 (8)	
C3	0.2764 (4)	−0.0662 (4)	1.07897 (19)	0.0257 (8)	
C4	0.1859 (4)	−0.1580 (3)	1.0060 (2)	0.0247 (8)	
C5	0.3312 (4)	0.0874 (4)	1.06546 (19)	0.0246 (8)	
H5	0.3908	0.1472	1.1134	0.030*	
C6	0.1489 (4)	−0.0948 (4)	0.92275 (19)	0.0245 (8)	
H6	0.0876	−0.1557	0.8757	0.029*	
C7	0.2984 (4)	0.1545 (4)	0.9806 (2)	0.0253 (8)	
C8	0.2032 (4)	0.0626 (4)	0.9075 (2)	0.0262 (8)	
C9	0.3508 (4)	0.3129 (4)	0.9647 (2)	0.0272 (8)	
H9	0.4124	0.3738	1.0116	0.033*	
C10	0.1628 (4)	0.1342 (4)	0.8220 (2)	0.0284 (8)	
H10	0.0984	0.0763	0.7743	0.034*	
C11	0.3125 (5)	0.3775 (4)	0.8816 (2)	0.0327 (8)	
H11	0.3488	0.4815	0.8726	0.039*	
C12	0.2174 (5)	0.2868 (4)	0.8089 (2)	0.0327 (9)	
H12	0.1924	0.3313	0.7525	0.039*	
N1	0.3053 (4)	−0.1348 (3)	1.16349 (17)	0.0273 (7)	
N2	0.1300 (4)	−0.3136 (3)	1.02231 (17)	0.0260 (7)	
O1	0.2918 (3)	−0.3464 (3)	1.25533 (14)	0.0361 (7)	
O2	0.1113 (3)	−0.5216 (3)	1.11523 (14)	0.0344 (7)	
H1N	0.354 (5)	−0.073 (5)	1.211 (2)	0.043 (10)*	
H2N	0.053 (6)	−0.366 (5)	0.975 (3)	0.059 (12)*	

### Atomic displacement parameters (Å^2^)

**Table:**

	*U*^11^	*U*^22^	*U*^33^	*U*^12^	*U*^13^	*U*^23^
C1	0.0346 (19)	0.0156 (17)	0.0319 (17)	0.0018 (13)	0.0036 (13)	−0.0041 (13)
C2	0.0318 (18)	0.0181 (17)	0.0319 (16)	0.0028 (13)	0.0028 (13)	0.0005 (13)
C3	0.0279 (18)	0.0199 (18)	0.0290 (16)	0.0010 (12)	0.0041 (12)	−0.0026 (13)
C4	0.0258 (18)	0.0128 (16)	0.0358 (17)	0.0013 (12)	0.0055 (13)	−0.0030 (12)
C5	0.0273 (17)	0.0139 (16)	0.0316 (16)	0.0028 (12)	0.0023 (12)	−0.0012 (12)
C6	0.0265 (17)	0.0172 (17)	0.0289 (15)	0.0001 (12)	0.0019 (12)	−0.0036 (12)
C7	0.0279 (18)	0.0137 (17)	0.0345 (16)	0.0027 (12)	0.0056 (13)	−0.0020 (13)
C8	0.0307 (18)	0.0146 (17)	0.0347 (17)	0.0018 (12)	0.0099 (13)	0.0023 (13)
C9	0.0272 (18)	0.0195 (18)	0.0347 (17)	−0.0006 (13)	0.0044 (13)	−0.0005 (13)
C10	0.0333 (19)	0.0197 (18)	0.0322 (16)	−0.0007 (13)	0.0052 (13)	−0.0029 (13)
C11	0.042 (2)	0.0199 (18)	0.0370 (18)	−0.0007 (14)	0.0081 (14)	0.0021 (13)
C12	0.044 (2)	0.0243 (19)	0.0300 (16)	0.0043 (14)	0.0064 (14)	0.0065 (13)
N1	0.0394 (17)	0.0154 (15)	0.0257 (14)	−0.0026 (11)	0.0013 (11)	−0.0027 (10)
N2	0.0342 (16)	0.0130 (14)	0.0297 (14)	−0.0001 (10)	0.0021 (11)	−0.0018 (10)
O1	0.0532 (16)	0.0191 (14)	0.0330 (13)	−0.0021 (10)	−0.0007 (10)	0.0033 (9)
O2	0.0459 (15)	0.0148 (13)	0.0400 (14)	−0.0017 (9)	0.0005 (10)	0.0008 (9)

### Geometric parameters (Å, º)

**Table:**

C1—O1	1.230 (4)	C6—H6	0.9300
C1—N1	1.356 (4)	C7—C9	1.417 (4)
C1—C2	1.518 (4)	C7—C8	1.432 (4)
C2—O2	1.240 (4)	C8—C10	1.425 (4)
C2—N2	1.349 (4)	C9—C11	1.366 (4)
C3—C5	1.378 (4)	C9—H9	0.9300
C3—N1	1.399 (4)	C10—C12	1.367 (4)
C3—C4	1.420 (4)	C10—H10	0.9300
C4—C6	1.364 (4)	C11—C12	1.423 (5)
C4—N2	1.404 (4)	C11—H11	0.9300
C5—C7	1.398 (4)	C12—H12	0.9300
C5—H5	0.9300	N1—H1N	0.91 (4)
C6—C8	1.412 (4)	N2—H2N	0.94 (4)
			
O1—C1—N1	123.8 (3)	C6—C8—C10	122.0 (3)
O1—C1—C2	119.7 (3)	C6—C8—C7	119.1 (3)
N1—C1—C2	116.5 (3)	C10—C8—C7	118.9 (3)
O2—C2—N2	122.8 (3)	C11—C9—C7	121.2 (3)
O2—C2—C1	119.4 (3)	C11—C9—H9	119.4
N2—C2—C1	117.7 (3)	C7—C9—H9	119.4
C5—C3—N1	121.7 (3)	C12—C10—C8	120.9 (3)
C5—C3—C4	119.8 (3)	C12—C10—H10	119.6
N1—C3—C4	118.5 (3)	C8—C10—H10	119.6
C6—C4—N2	121.0 (3)	C9—C11—C12	120.4 (3)
C6—C4—C3	120.7 (3)	C9—C11—H11	119.8
N2—C4—C3	118.3 (3)	C12—C11—H11	119.8
C3—C5—C7	120.8 (3)	C10—C12—C11	120.1 (3)
C3—C5—H5	119.6	C10—C12—H12	120.0
C7—C5—H5	119.6	C11—C12—H12	120.0
C4—C6—C8	120.4 (3)	C1—N1—C3	124.6 (3)
C4—C6—H6	119.8	C1—N1—H1N	117 (2)
C8—C6—H6	119.8	C3—N1—H1N	118 (2)
C5—C7—C9	122.2 (3)	C2—N2—C4	124.2 (3)
C5—C7—C8	119.2 (3)	C2—N2—H2N	119 (3)
C9—C7—C8	118.6 (3)	C4—N2—H2N	117 (3)
			
O1—C1—C2—O2	1.7 (5)	C5—C7—C8—C10	177.3 (3)
N1—C1—C2—O2	−176.8 (3)	C9—C7—C8—C10	−0.8 (4)
O1—C1—C2—N2	179.3 (3)	C5—C7—C9—C11	−178.1 (3)
N1—C1—C2—N2	0.8 (4)	C8—C7—C9—C11	−0.1 (5)
C5—C3—C4—C6	−1.3 (5)	C6—C8—C10—C12	−179.8 (3)
N1—C3—C4—C6	177.9 (3)	C7—C8—C10—C12	1.5 (5)
C5—C3—C4—N2	−179.3 (3)	C7—C9—C11—C12	0.4 (5)
N1—C3—C4—N2	−0.1 (4)	C8—C10—C12—C11	−1.3 (5)
N1—C3—C5—C7	−179.0 (3)	C9—C11—C12—C10	0.4 (5)
C4—C3—C5—C7	0.2 (5)	O1—C1—N1—C3	178.1 (3)
N2—C4—C6—C8	179.0 (2)	C2—C1—N1—C3	−3.5 (4)
C3—C4—C6—C8	1.1 (5)	C5—C3—N1—C1	−177.6 (3)
C3—C5—C7—C9	179.2 (3)	C4—C3—N1—C1	3.2 (5)
C3—C5—C7—C8	1.2 (5)	O2—C2—N2—C4	179.7 (3)
C4—C6—C8—C10	−178.4 (3)	C1—C2—N2—C4	2.2 (4)
C4—C6—C8—C7	0.3 (5)	C6—C4—N2—C2	179.5 (3)
C5—C7—C8—C6	−1.4 (4)	C3—C4—N2—C2	−2.6 (5)
C9—C7—C8—C6	−179.5 (3)		

### Hydrogen-bond geometry (Å, º)

**Table:**

*D*—H···*A*	*D*—H	H···*A*	*D*···*A*	*D*—H···*A*
N1—H1*N*···O1^i^	0.91 (4)	2.27 (4)	2.866 (3)	122 (3)
N2—H2*N*···O2^ii^	0.94 (4)	1.90 (4)	2.843 (4)	176 (4)

Symmetry codes: (i) −*x*+1/2, *y*+1/2, −*z*+5/2; (ii) −*x*, −*y*−1, −*z*+2.
